# Evaluation of Bronchoalveolar Lavage Fluid Cytokines as Biomarkers for Invasive Pulmonary Aspergillosis in At-Risk Patients

**DOI:** 10.3389/fmicb.2017.02362

**Published:** 2017-11-29

**Authors:** Samuel M. Gonçalves, Katrien Lagrou, Cláudia S. Rodrigues, Cláudia F. Campos, Leticia Bernal-Martínez, Fernando Rodrigues, Ricardo Silvestre, Laura Alcazar-Fuoli, Johan A. Maertens, Cristina Cunha, Agostinho Carvalho

**Affiliations:** ^1^Life and Health Sciences Research Institute (ICVS), School of Medicine, University of Minho, Braga, Portugal; ^2^ICVS/3B's—PT Government Associate Laboratory, Braga/Guimarães, Portugal; ^3^Department of Microbiology and Immunology, KU Leuven, Leuven, Belgium; ^4^Department of Laboratory Medicine and National Reference Center for Medical Mycology, University Hospitals Leuven, Leuven, Belgium; ^5^Mycology Reference Laboratory, National Centre for Microbiology, Instituto de Salud Carlos III, Madrid, Spain; ^6^Department of Hematology, University Hospitals Leuven, Leuven, Belgium

**Keywords:** invasive pulmonary aspergillosis, biomarkers, cytokines/chemokines, bronchoalveolar lavage, fungal diagnostics

## Abstract

**Background:** Invasive pulmonary aspergillosis (IPA) is an infection that primarily affects immunocompromised hosts, including hematological patients and stem-cell transplant recipients. The diagnosis of IPA remains challenging, making desirable the availability of new specific biomarkers. High-throughput methods now allow us to interrogate the immune system for multiple markers of inflammation with enhanced resolution.

**Methods:** To determine whether a signature of alveolar cytokines could be associated with the development of IPA and used as a diagnostic biomarker, we performed a nested case-control study involving 113 patients at-risk.

**Results:** Among the 32 analytes tested, IL-1β, IL-6, IL-8, IL-17A, IL-23, and TNFα were significantly increased among patients with IPA, defining two clusters able to accurately differentiate cases of infection from controls. Genetic variants previously reported to confer increased risk of IPA compromised the production of specific cytokines and impaired their discriminatory potential toward infection. Collectively, our data indicated that IL-8 was the best performing cytokine, with alveolar levels ≥904 pg/mL predicting IPA with elevated sensitivity (90%), specificity (73%), and negative predictive value (88%).

**Conclusions:** These findings highlight the existence of a specific profile of alveolar cytokines, with IL-8 being the dominant discriminator, which might be useful in supporting current diagnostic approaches for IPA.

## Introduction

Invasive pulmonary aspergillosis (IPA) is a life-threatening infection caused predominantly by the opportunistic fungus *Aspergillus fumigatus* (Segal, 2009). It is commonly diagnosed among patients with immunological deficits, namely those with hematologic malignancies during chemotherapy or undergoing solid organ or allogeneic hematopoietic cell transplantation (Kontoyiannis et al., 2010; Pagano et al., 2010; Pappas et al., 2010). There are currently no licensed vaccines, and despite improved diagnosis and therapy, management of IPA remains challenging, with mortality rates of infected patients exceeding 30% (Bitar et al., 2014; Maertens et al., 2016). Given the variable risk of infection and its clinical outcome among patients with comparable predisposing clinical and microbiological factors, susceptibility to IPA is thought to rely largely on genetic predisposition (Cunha et al., 2013; Camargo and Husain, 2014).

Early diagnosis of IPA is critically required to decrease morbidity and mortality, particularly in vulnerable populations of immunocompromised patients, since the delayed initiation of antifungal therapy may contribute to fatal outcomes (Arvanitis et al., 2015). However, definitive diagnoses are challenging, typically because traditional diagnostic tools, such as histology and culture, are often difficult to obtain, with relatively low sensitivities, particularly in patients under antifungal prophylaxis. The incorporation of fungal surrogate markers, such as galactomannan, into clinical algorithms has facilitated diagnostic-driven strategies in at-risk patients (Morrissey et al., 2013). Notwithstanding, treatment of IPA remains mostly empirical based on a high index of suspicion, combined with non-specific clinical signs and symptoms, and radiological findings (Kozel and Wickes, 2014). This results in an excessive prescription of antifungal drugs, ultimately associated with a remarkable economic burden to the healthcare systems, highlighting therefore a pressing demand for new and improved diagnostic methods for IPA (Oliveira-Coelho et al., 2015).

Our current view of the pathogenesis of IPA identifies the concerted action of the ciliated epithelium and cells of the innate immune system, including resident alveolar macrophages and dendritic cells, and recruited inflammatory cells, as the first line of defense against inhaled fungal spores (Espinosa and Rivera, 2016). These cells express a large repertoire of immune receptors that sense pathogen motifs and drive the secretion of cytokines and chemokines that control innate and adaptive immune responses (Carvalho et al., 2012b). Because of their production in response to infection, specific cytokines and chemokines, such as interleukin (IL)-6, IL-8, and IL-10, have been reported at higher concentrations in the serum of patients suffering from IPA compared to controls (Chai et al., 2010a,b; Reikvam et al., 2012; Ceesay et al., 2016; Shen et al., 2016). Remarkably, IL-6 was also reported to discriminate cases of IPA from *Pneumocystis* pneumonia in pediatric oncology patients (Shen et al., 2016), thereby raising the appealing possibility for cytokine detection in differential fungal diagnostics. Besides their diagnostic value, certain cytokines have been endowed with prognostic significance. For example, persistently elevated levels of circulating IL-6 and IL-8 were identified as important early predictors of adverse outcomes in IPA (Chai et al., 2010a).

The genetic profile of the patient is regarded as a critical factor contributing to the risk of IPA (Cunha et al., 2013). Accordingly, variants in cytokine and chemokine genes impairing their expression or functional activity have been disclosed as major determinants of susceptibility to infection (Mezger et al., 2008; Wojtowicz et al., 2014; Cunha et al., 2017). Likewise, genetic variants affecting the β-glucan receptor dectin-1, and known to predispose to IPA, triggered a significant defect in cytokine production following experimental fungal infection (Cunha et al., 2010). Although the list of circulating cytokines and chemokines that correlate with the risk and clinical outcome of IPA continues to grow, a systematic profiling of alveolar cytokines during infection and the evaluation of their diagnostic significance has never been performed.

To determine whether alveolar cytokines were endowed with diagnostic potential in IPA, we measured 32 analytes in bronchoalveolar lavage (BAL) samples from a nested case-control study involving 113 patients at-risk of infection. We found that a subset of alveolar cytokines could significantly discriminate cases of infection from controls. In addition, we identified two distinct clusters of highly correlated cytokines that were differentially expressed between cases of IPA and controls. The diagnostic performance of individual or clustered cytokines was found to depend on the genetic background of the patient. Taken together, the results presented herein provide crucial insights into the pulmonary immune profile of patients with IPA and disclose individual and clustered cytokines that may serve as important diagnostic adjuvants in combination with classical diagnostic methods.

## Materials and methods

### Patients and sample collection

BAL and serum samples were collected from hospitalized adult patients (≥18 years of age) during routine diagnostic workup following suspicion of infection at the Leuven University Hospitals, Leuven, Belgium. The demographic and clinical characteristics of the patients enrolled are summarized in Table 1. Fifty-seven cases of “probable” or “proven” IPA were identified according to the revised standard criteria from the European Organization for Research and Treatment of Cancer/Mycology Study Group (EORTC/MSG) (De Pauw et al., 2008). The control group included patients with no evidence for the presence of *Aspergillus* spp. in the BAL (negative culture and galactomannan testing). Patients with “possible” disease were excluded from the study and no mold-active drugs were administered by the treating physician(s) before sample collection. This study was approved and carried out in accordance with recommendations of the Ethics Subcommittee for Life and Health Sciences of the University of Minho, Portugal, and the Ethics Committee of the University Hospitals of Leuven, Belgium. Written informed consent was obtained from all subjects in accordance with the Declaration of Helsinki.

**Table 1:** Baseline characteristics of patients enrolled in the study.

**Variables**	**IPA (*n* = 57)**	**No IPA (*n* = 56)**	***P*-value**
**Age, no (%)**			
≤ 50 years	9 (15.8)	14 (25.0)	0.25
>50 years	48 (84.2)	42 (75.0)	
**Gender, no (%)**			
Female	26 (45.6)	24 (42.9)	0.85
Male	31 (54.4)	32 (57.1)	
**Underlying disease, no. (%)**			
SOT^†^	12 (21.1)	20 (35.7)	0.13
Allogeneic SCT	10 (17.5)	12 (21.4)	
Acute leukemia	10 (17.5)	10 (17.9)	
Chronic lymphoproliferative diseases	10 (17.5)	7 (12.5)	
Influenza A (H1N1)	6 (10.5)	0 (0.0)	
Chronic lung diseases	4 (7.0)	1 (1.8)	
Solid tumors	2 (3.5)	1 (1.8)	
Other	3 (5.3)	5 (8.9)	
**Neutrophil counts, × 10^3^ cells/μL (range)**	5.6 (0.0–24.8)	5.2 (1.9–17.3)	0.40
**GMI, mean (range)**	5 (1.0–6.9)	0.2 (0.1–0.4)	<0.001
**Other pathogens detected in BAL fluid**			
Bacteria	8 (14.0)	8 (14.3)	0.42
Viruses	22 (38.6)	10 (17.9)	
Fungi^‡^	6 (10.5)	3 (5.4)	

*SOT, solid organ transplantation; SCT, stem-cell transplantation; GMI, galactomannan index; BAL, bronchoalveolar lavage; P-values were calculated by Fisher's exact probability t-test or Student's t-test for continuous variables*.

† *The study included 32 patients who received an SOT from lung (n = 27), kidney (n = 3), and liver (n = 2). Among those, 12 were diagnosed with IPA (lung, n = 8; kidney, n = 2; and liver, n = 2)*.

‡ *Among fungi, Pneumocystis spp. was detected in five patients with IPA and three controls*.

### BAL fluid collection

BAL specimens were collected using a flexible fiberoptic bronchoscope following local anesthesia with 2% lidocaine (Xylocaine), when infection was clinically suspected. Samples were obtained by instillation of a pre-warmed 0.9% sterile saline solution (20 mL twice). The sampling area was determined based on the localization of lesion on chest imaging (X-ray or computed tomography scan). BAL specimens with comparable recovery rates were used. All samples were stored at −80°C until use.

### Galactomannan testing

The Platelia *Aspergillus* EIA (Bio-Rad, Marnes-la-Coquette, France) was used during routine microbiological workup to detect the presence of galactomannan on uncentrifuged BAL specimens, as described (D'Haese et al., 2012). The enzyme immunoassay data was expressed as galactomannan index (GMI).

### ELISA

Cytokines were quantified in BAL and serum samples using customized Human Premixed Multi-Analyte Kits (R&D Systems, MN, USA). All cytokine determinations were performed in duplicates, and concentrations were reported in pg/mL.

### Single nucleotide polymorphism (SNP) selection and genotyping

SNPs were selected based on previously reported associations with increased risk of developing IPA (Cunha et al., 2010, 2014) that were independently validated (Chai et al., 2011; Fisher et al., 2017). Genomic DNA was isolated from whole blood using the QIAcube automated system (Qiagen, Hilden, Germany). Genotyping of rs2305619 in *PTX3* and rs16910526 in *CLEC7A* (dectin-1) was performed using KASPar assays (LGC Genomics, Hertfordshire, UK) in an Applied Biosystems 7500 Fast Real-Time PCR system (Thermo Fisher Scientific, MA, USA), according to the manufacturer's instructions. Mean call rate for the SNPs was >98%. Quality control for the genotyping results was achieved with negative controls and randomly selected samples with known genotypes.

### Statistical analysis

Statistical analyses were performed using the Prism Version 7.0 (GraphPad Software) or R version 3.4.1. *P*-values < 0.05 were considered statistically significant, unless indicated otherwise. The concentrations for each cytokine in BAL and serum specimens were compared between cases of IPA and controls using either the Student's *t*-test or the non-parametric Mann-Whitney *U*-test, following the Shapiro-Wilk normality test. Cytokine levels were expressed as log_10_ pg/mL, and data were represented as mean ± SEM. Categorical variables were compared using the Fisher's exact test.

Random Forest Analysis (RFA) was used to rank cytokine levels in importance toward phenotype prediction. Calculations were carried out tree-by-tree as the random forest was constructed and were run >100 times to assess the robustness of ordering using the *randomForest* package for R. To identify sets of cytokines whose expression levels were correlated among patients, an unsupervised hierarchical clustering was applied using log_2_ transformed cytokine values. A heatmap was produced using the Morpheus platform (Broad Institute, MA, USA) using average linkage on a similarity matrix derived by Pearson moment correlations between patients (vertical clusters) or cytokines (horizontal clusters). Cytokine levels in clusters 1 (C1) and 2 (C2) for a given patient were combined by adding contributions based on the log_2_ transformed levels of the individual cytokines, as described (Yan et al., 2011). The median level for each cluster for all the patients was determined and patients were evaluated as to whether they were high (“Hi”) or low (“Lo”) for that cytokine cluster based on their individual value relative to the median.

The discriminatory ability of each cytokine was measured as the area under the receiving operating characteristic curve (AUC^ROC^). Sensitivity, specificity, positive predictive value (PPV), negative predictive value (NPV), and Youden index values were computed to evaluate the cytokine thresholds with the highest discriminatory power. The net reclassification index (NRI) was used to compare the performance of the identified cut-off levels of specific cytokines in BAL with other variables, as indicated.

## Results

### Alveolar immune profiling reveals a subset of cytokines differentially expressed in IPA

**Figure 1:**
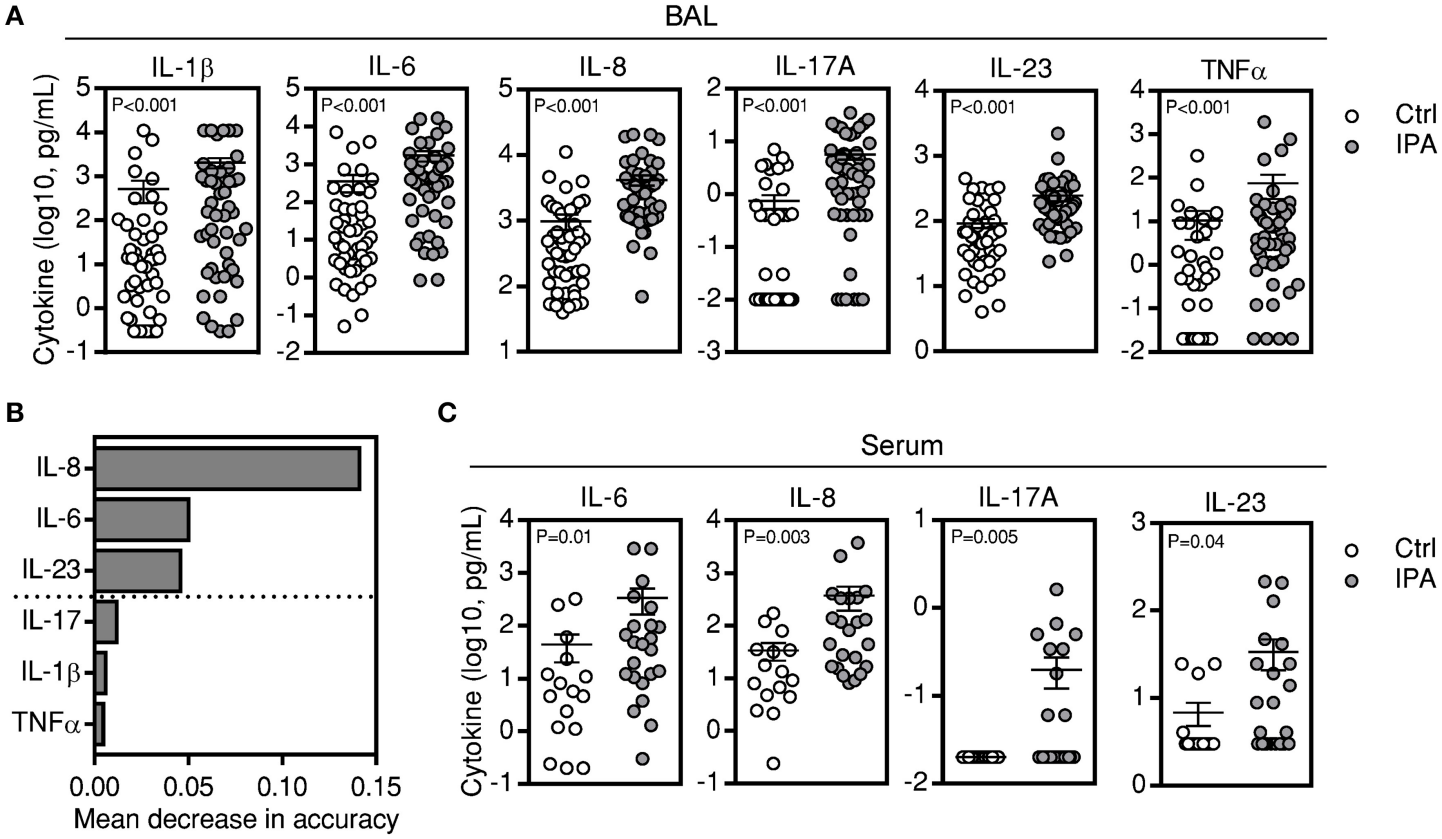
A subset of BAL cytokines is differentially expressed in IPA. **(A)** Levels of cytokines present in the BAL of patients with IPA compared with controls (Ctrl). Data are presented as mean ± SEM values. **(B)** Ranking of cytokines by their relative importance in discriminating cases of IPA from controls using RFA. The horizontal axis represents the average decrease in classification accuracy, and bars indicate the relative importance of each individual cytokine to discrimination. The dashed line divides cytokines at the mean value of decrease in accuracy and defines the number of cytokines required for maximum classification accuracy. **(C)** Levels of cytokines present in the sera of patients with IPA compared with controls (Ctrl). Data are presented as mean ± SEM values.

To gain insight into the alveolar cytokine profile associated with the development of IPA, we compared the levels of single cytokines in the BAL from patients diagnosed with IPA and matched controls. BAL samples from 48 patients with IA and 48 matched controls were available and analyzed for cytokine levels. From the initial set of 32 analytes tested, we found that patients with IPA displayed significantly higher levels of IL-1β, IL-6, IL-8, IL-17A, IL-23, and TNFα after adjusting for multiple comparisons (Figure 1A; Table S1). Among the differentially expressed BAL cytokines, random forest analysis (RFA) revealed that IL-8, IL-6, and IL-23 best differentiated between cases of IPA and controls, whereas the remaining cytokines displayed an inconsistent contribution to discrimination (Figure 1B). Levels of IL-8 were the dominant discriminator, although the full set distinguished cases from controls. No differences were observed in the BAL cytokine levels according to the neutropenic status of the patients with IPA (Figure S1). Consistent with the BAL data, and despite the globally lower absolute cytokine concentrations detected in the serum, circulating IL-6, IL-8, IL-17A, and IL-23 were also significantly increased among patients with IPA (only IL-17A remained significant after adjustment for multiple comparisons) (Figure 1C; Table S2).

### Two-dimensional cluster analysis defines two BAL cytokine sets related with IPA

**Figure 2:**
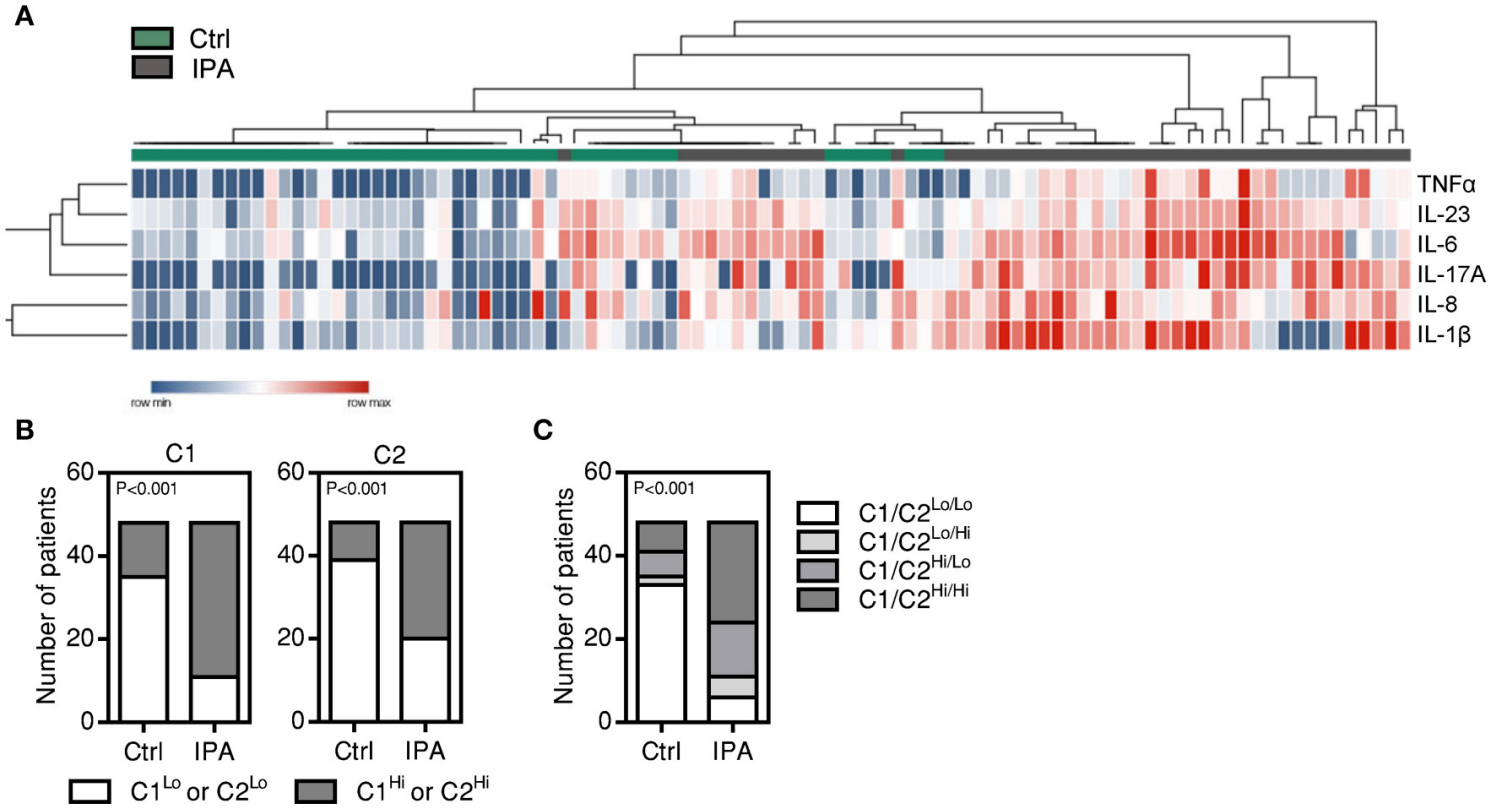
Cluster analysis reveals two groups of highly expressed BAL cytokines in IPA. **(A)** Unsupervised hierarchical clustering for IL-1β, IL-6, IL-8, IL-17A, IL-23, and TNFα. Expression levels of individual cytokines are represented by shades of blue to red in the heatmap, with highest values in dark red and lowest values in dark blue. The top dendrogram illustrates the separate clustering of cases of IPA (indicated by gray boxes) from controls (Ctrl) (indicated by green boxes). The left dendrogram illustrates the identification of two cytokine clusters: C1 (TNFα, IL-23, IL-6, and IL-17A) and C2 (IL-8 and IL-1β). **(B)** Number of patients presenting low and high values of C1 (C1^Lo^ and C1^Hi^, respectively) and C2 cytokines (C2^Lo^ and C2^Hi^, respectively) among controls (Ctrl) and cases of IPA. **(C)** Number of patients with combined information on the levels of C1 and C2 cytokines among controls (Ctrl) and cases of IPA. Four categories are indicated: C1^Lo^/C2^Lo^, C1^Lo^/C2^Hi^, C1^Hi^/C2^Lo^, C1^Hi^/C2^Hi^.

Previous studies have shown that cytokines can be released in clusters, and this coordinated release may reflect common regulatory mechanisms (Ter Horst et al., 2016). Unsupervised hierarchal clustering analysis revealed that patients with IPA clustered separately from controls based on the levels of individual BAL cytokines (Figure 2A). In addition, two cytokine sets able to differentiate cases of IPA from controls were defined: cluster 1 (C1), including IL-6, IL-17A, IL-23, and TNFα, and cluster 2 (C2), which included IL-1β and IL-8. Further supporting these results, higher median levels of C1 and C2 cytokines in the BAL were more frequently observed in patients with IPA (37 C1^Hi^ and 28 C2^Hi^ patients out of 48) than controls (13 C1^Hi^ and 9 C2^Hi^ patients out of 48) (Figure 2B). The combined analysis of C1 and C2 clusters also revealed that higher levels of cytokines from both clusters were more common among patients with IPA compared to controls (24 C1^Hi^/C2^Hi^ patients out of 48) (Figure 2C).

### Positivity for galactomannan influences the levels of BAL cytokines

**Figure 3:**
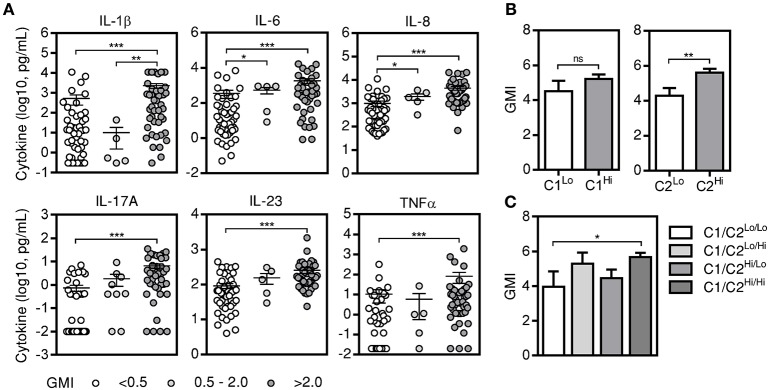
BAL cytokine levels depend on galactomannan positivity. **(A)** Levels of alveolar cytokines in patients with negative (<0.5) and different ranges of GMI positivity (0.5–2.0 and >2.0). Data are presented as mean ± SEM values; ^***^p < 0.001; ^**^p < 0.01; ^*^p < 0.05. **(B)** GMI of patients displaying low and high values of C1 (C1^Lo^ and C1^Hi^, respectively) and C2 cytokines (C2^Lo^ and C2^Hi^, respectively). Data are presented as mean ± SEM; ^**^p < 0.01. **(C)** GMI of patients with combined information on the levels of C1 and C2 cytokines. Four categories are indicated: C1^Lo^/C2^Lo^, C1^Lo^/C2^Hi^, C1^Hi^/C2^Lo^, C1^Hi^/C2^Hi^. Data are presented as mean ± SEM; ^*^p < 0.05.

The detection of galactomannan in BAL specimens has been advocated as a sensitive test for diagnosing IPA, particularly when interpreted in combination with clinical and radiological findings (D'Haese et al., 2012). To understand whether galactomannan in the BAL could influence the ability of cytokines to discriminate cases of IPA from controls, we analyzed individual and clustered cytokine data according to GMI: <0.5 (identifying controls), 0.5–2.0 (representing the most commonly used cut-off values for positivity), and >2.0. We found that patients with high, but not intermediate, GMI displayed significantly elevated concentrations of BAL cytokines compared to controls (Figure 3A). Importantly, only IL-6 and IL-8 retained their discriminatory potential for IPA in patients with intermediate GMI. In addition, by comparing the expression of cytokine clusters, we found that the C1 profile was not influenced by the GMI (Figure 3B). Instead, C2^Hi^ patients displayed significantly higher mean values of GMI than C2^Lo^ patients. Consistent with this, patients with a combined C1^Hi^/C2^Hi^ cytokine profile presented higher mean values of GMI than patients belonging to the C1^Lo^/C2^Lo^ category (5.7 vs. 4.0) (Figure 3C).

### Genetic variants conferring risk of IPA impair the discriminatory ability of BAL cytokines

**Figure 4:**
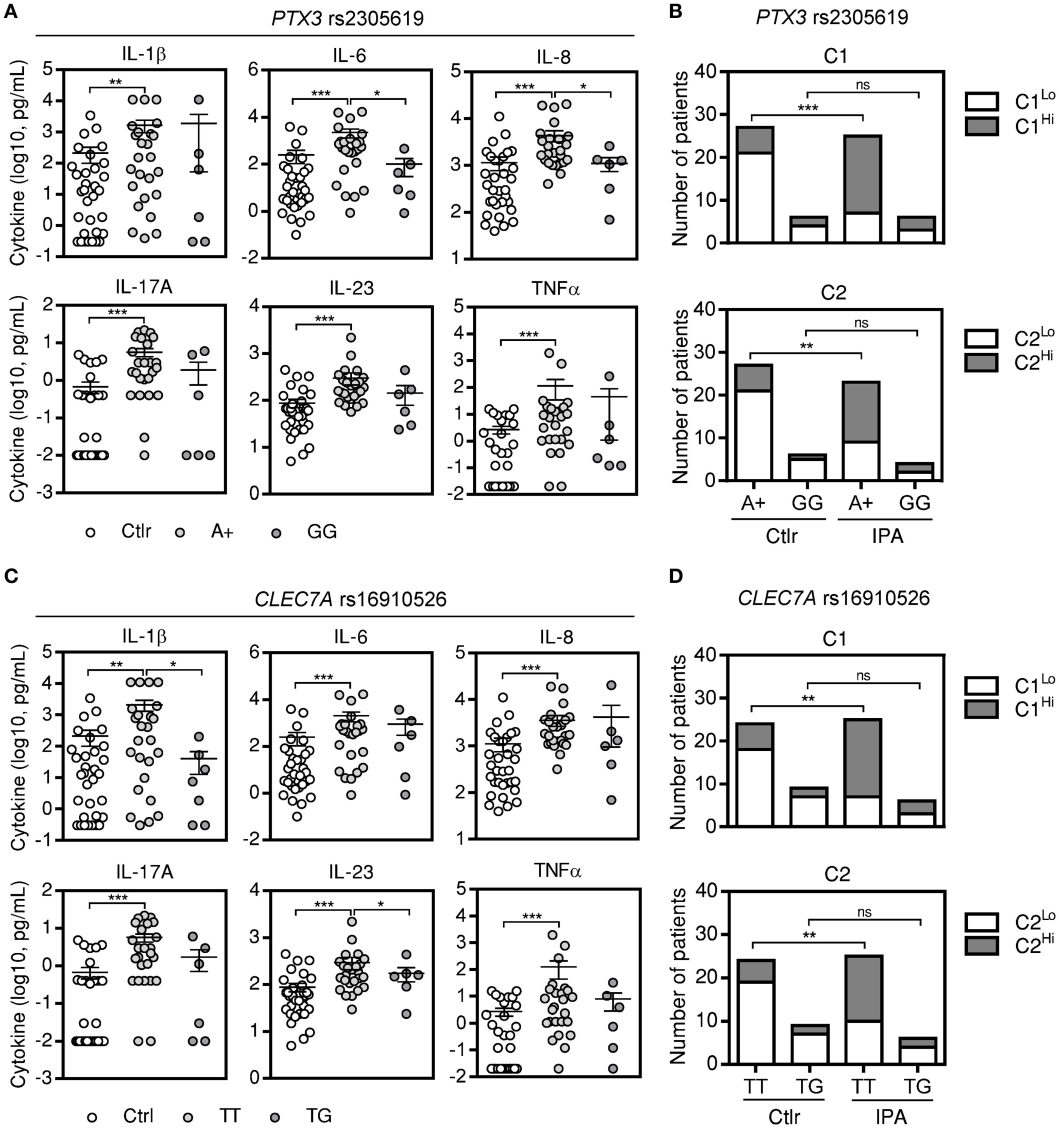
Genetic variants conferring risk to IPA influence the levels of BAL cytokines. **(A)** Levels of alveolar cytokines in patients with IPA carrying different genotypes at rs2305619 in *PTX3*. A+ indicates combined AA and AG genotypes. Data are presented as mean ± SEM values; ^***^p < 0.001, ^**^p < 0.01, ^*^p < 0.05. **(B)** Distribution of low and high values of C1 and C2 in IPA and controls (Ctrl) in the presence of different genotypes at rs2305619 in *PTX3*. Vertical axis represents the number of patients with low or high values of C1 and C2 while horizontal axis represents the different genotypes; ^***^p < 0.001, ^**^p < 0.01. **(C)** Levels of alveolar cytokines in patients with IPA carrying different genotypes at rs16910526 in *CLEC7A*. Data are presented as mean ± SEM values; ^***^p < 0.001, ^**^p < 0.01, ^*^p < 0.05. **(D)** Distribution of low and high values of C1 and C2 in IPA and controls (Ctrl) in the presence of different genotypes at rs16910526 in *CLEC7A*. Vertical axis represents the number of patients with low or high values of C1 and C2 while horizontal axis represents the different genotypes; ^**^p < 0.01.

A number of studies has disclosed an important contribution of host genetics in defining susceptibility to IPA (Cunha et al., 2013). However, with a few exceptions, their consequences to cytokine production remain elusive. We analyzed cytokine data according to the genotypes of two of the most robust genetic markers for IPA identified to date, rs2305619 in *PTX3* (Cunha et al., 2014, 2015; Wojtowicz et al., 2015) and rs16910526 in *CLEC7A* (Cunha et al., 2010; Chai et al., 2011). We found that patients harboring AA or AG (referred to as A+) genotypes at rs2305619 retained increased levels of BAL cytokines when compared to controls (Figure 4A). However, the production of IL-6 and IL-8 was significantly impaired by the presence of the high-risk GG genotype. Accordingly, cluster analysis revealed differences in the expression of cytokines between cases of IPA and controls with the A+, but not GG, genotypes (Figure 4B). Similar findings were observed for the rs16910526 variant in *CLEC7A*, with increased levels of BAL cytokines detected in patients carrying the TT genotype compared to controls (Figure 4C). Production of IL-1β and IL-23 was instead impaired among patients with IPA carrying the high-risk TG genotype. As expected, expression of clustered cytokines was different between cases and controls with the TT, but not TG, genotypes (Figure 4D). Similar results were obtained for combined cluster analysis (Figure S2). No differences were noted in cytokine levels among control subjects according to the *PTX3* or *CLEC7A* genotypes (data not shown).

### BAL cytokines represent potentially useful diagnostic biomarkers for IPA

**Figure 5:**
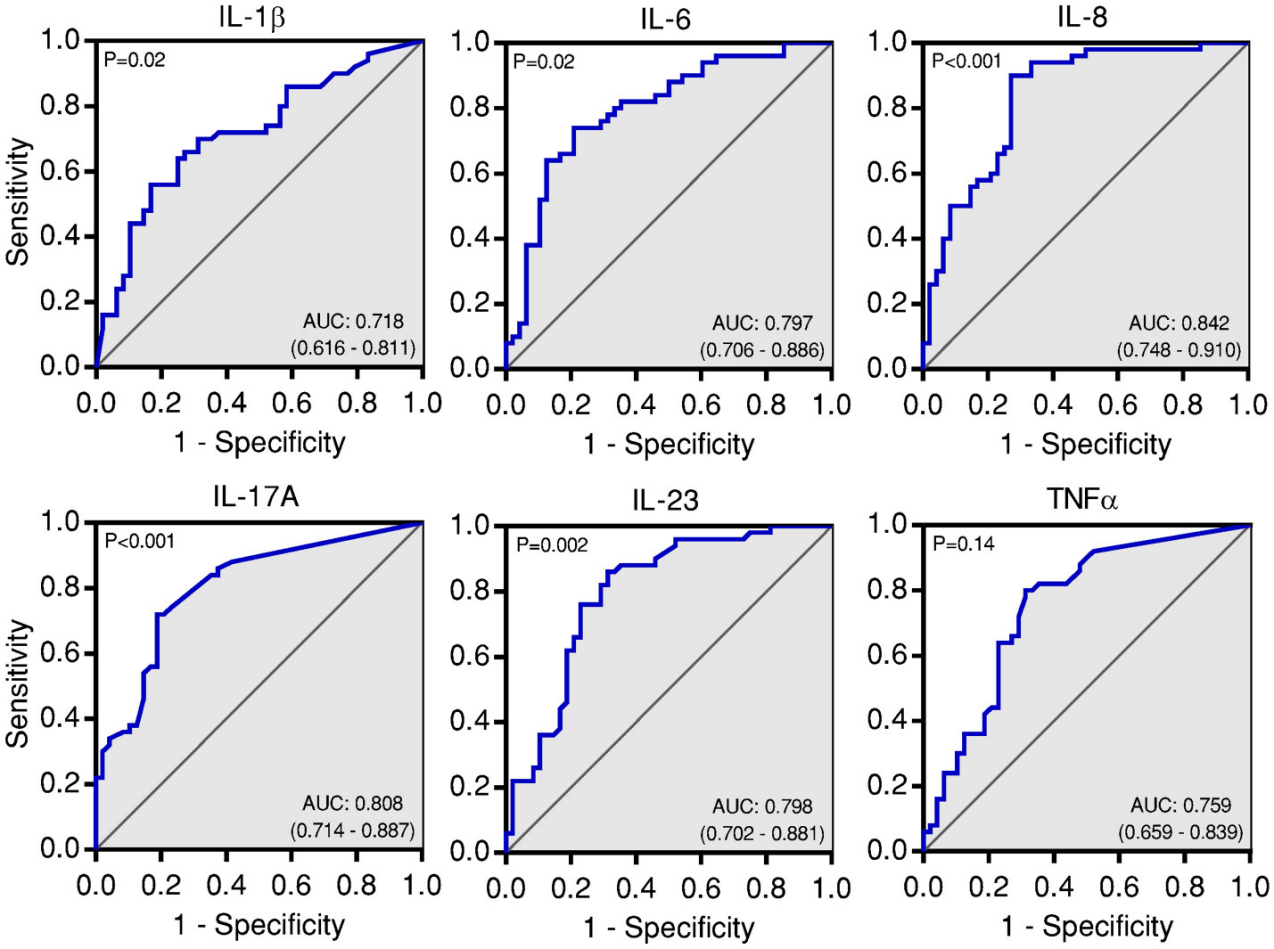
BAL cytokines accurately predict the development of IPA. Area under the receiver operating characteristic curve (AUC^ROC^) analysis for each BAL cytokine demonstrating sensitivity as a function of one-specificity for the prediction of IPA.

To corroborate the utility of BAL cytokines for the diagnosis of IPA, we analyzed the AUC^ROC^ for each cytokine (Figure 5; Table 2). As expected, all cytokines tested, except for TNFα, demonstrated reasonable sensitivity and specificity. IL-8 was the best performing analyte (AUC^ROC^ = 0.84; 95% CI, 0.75–0.91; P < 0.001), with a cut-off level of IL-8 ≥904 pg/mL associated with 90% sensitivity, 73% specificity, 78% PPV, and 88% NPV. Total accuracy conferred by alveolar IL-8 was 82%: five patients with IPA presented values below the cut-off, whereas 13 control subjects displayed instead values above the cut-off.

**Table 2:** Performance of BAL cytokines as diagnostic biomarkers for IPA.

**Cytokine**	**Cut-off^†^**	**Sensitivity**	**Specificity**	**PPV**	**NPV**	**NRI**
		**(95% CI)**				
IL-1β	27.1	70 (55–83)	68 (55–81)	70 (60–79)	69 (58–78)	0.34
IL-6	89.8	74 (63–85)	79 (68–89)	78 (67–87)	73 (63–81)	0.51
IL-8	904	90 (81–98)	73 (60–85)	78 (68–85)	88 (75–94)	0.63
IL-17A	0.66	72 (58–84)	81 (70–90)	80 (68–88)	74 (64–82)	0.53
IL-23	103	76 (66–90)	77 (67–90)	78 (67–86)	76 (65–84)	0.53
TNF-α	0.94	80 (70–90)	69 (55–81)	73 (63–81)	77 (65–86)	0.49

† *Cut-off values of cytokines are expressed as pg/mL. Statistically-derived optimal cut-off was determined by Youden's index (maximum sensitivity and specificity given by the inflection point of the AUC^ROC^). The net reclassification index (NRI) was used to compare the performance of each cytokine cut-off with the known diagnosis of IPA. IL, interleukin; TNF, tumor necrosis factor; PPV, positive predictive value; NPV, negative predictive value*.

Consistent with the results pointing to a detrimental role of specific risk genotypes for IPA regarding cytokine production, we observed that the high-risk GG genotype at rs2305619 in *PTX3* led to a decrease of 23% sensitivity for IL-8 and 29% specificity for IL-6, hindering their performance in identifying cases (AUC^ROC^ = 0.67; 95% CI, 0.33–0.97; *P* = 0.70) and controls (AUC^ROC^ = 0.58; 95% CI, 0.22–0.89; *P* = 0.80), respectively (Table S3). Likewise, the high-risk TG genotype at rs2305619 in *CLEC7A* precluded the ability of IL-1β (AUC^ROC^ = 0.63; 95% CI, 0.37–0.89; *P* = 0.51) and IL-17A (AUC^ROC^ = 0.69; 95% CI, 0.41–0.94; *P* = 0.21) to identify controls by impairing specificity by 12 and 14%, respectively. Collectively, these results highlight IL-8 as the most relevant discriminator between cases of IPA and controls and highlight the genetic background of the patients as a critical factor to consider when evaluating the diagnostic performance of host-derived biomarkers.

## Discussion

The initiation of an efficient antifungal immune response depends on a complex set of signals circulating within the microenvironment, including cytokines and chemokines (Hohl, 2017). Understanding how each of these pathways is regulated is essential to uncover the molecular and cellular processes underlying the pathogenesis of IPA, and may also offer crucial insights toward the identification of molecules that correlate strongly with infection and the network of signals that could be therapeutically targeted.

In our exploratory study, we quantified the alveolar levels of 32 analytes in patients with IPA and matched controls, and analyzed these findings based on individual or analytically clustered sets of mediators. Our data provide strong evidence for an alveolar cytokine profile that is differentially expressed in patients with IPA. The observed inflammatory phenotype is largely attributable to IPA rather than to a general response to infection since events of viral, bacterial and even *Pneumocystis* pneumonia were diagnosed within the control group (Table 1). This is consistent with the recent finding that cytokine production is organized around the physiological response toward specific pathogens rather than through specific immune pathways (Li et al., 2016).

Prior studies of biomarker evaluation have mostly addressed candidate cytokines, reporting altered circulating levels to be correlated with the development of IPA (Camargo and Husain, 2014). In addition, the generation of specific cytokine-producing T-cells in response to *A. fumigatus* antigens have also been exploited as a potential immunodiagnostic approach (Potenza et al., 2013). Our study is among the first demonstrations that alveolar cytokines may also be relevant biomarkers for the diagnosis of IPA, regardless of the neutrophil counts at the time of diagnosis. In fact, we found that BAL cytokines performed better than the circulating counterpart, likely because they mirror more accurately the pathophysiology of IPA, with cytokines being produced mostly by immune cells at the site of infection rather than in the periphery (Kontoyiannis, 2011). Of note, and although galactomannan has been suggested to suppress cytokine responses (Chai et al., 2009), patients with high GMI displayed increased levels of alveolar cytokines than controls, demonstrating that the combination of relevant cytokines with fungal surrogate markers may improve our capacity to predict disease outcome.

Within the differential alveolar profile, we identified several inflammatory cytokines with well-known roles in antifungal immune responses, such as IL-1β, IL-6, IL-8, and TNFα (Becker et al., 2015). Among these, IL-8 was the best performing analyte with a total accuracy above 80%. These findings are supported by a recent report on a small patient cohort that also disclosed alveolar IL-8 as a potentially useful biomarker for IPA (Heldt et al., 2017). This is in accordance with its role as a central regulatory cytokine produced mainly by alveolar macrophages and epithelial cells early after infection to coordinate the recruitment of inflammatory cells (Balloy et al., 2008). Interestingly, and despite previous reports pointed to a minor contribution of T helper (Th)17 responses to *A. fumigatus* in cellular models of infection (Chai et al., 2010b), IL-17A and IL-23 in the BAL were also upregulated, supporting a role for the Th17 pathway during IPA (Zelante et al., 2007).

The risk of IPA and its clinical outcome vary significantly even among patients with similar predisposing clinical conditions and microbiological exposure (Cunha et al., 2013). Although a number of genetic variants in cytokine genes have also been associated with the development of IPA (Loeffler et al., 2010), rs2305619 in *PTX3* and rs16910526 in *CLEC7A* have been recently proposed as the most robust genetic markers identified to date (Fisher et al., 2017). PTX3 is an important fluid-phase pattern recognition molecule with ancestral antibody-like properties that recognizes and interacts with *A. fumigatus* to exert critical roles in antifungal innate immunity (Garlanda et al., 2002). Remarkably, IL-6 and IL-8 were influenced by the presence of the risk genotype at rs2305619, known to impair the expression of PTX3 in the lung (Cunha et al., 2014). This may suggest that, similar to other opsonins such as L-ficolin (Bidula et al., 2015), PTX3-mediated conidia opsonization is critically required to potentiate IL-8 secretion in the lung. Whatever the mechanism(s), the functional crosstalk between molecules with opsonic activity and other inflammatory mediators in antifungal immunity remains to be thoroughly explored. In any case, it is not surprising that alveolar, but not serum, PTX3 has been disclosed as a valuable early marker for microbiologically-confirmed pneumonia (Mauri et al., 2014).

The levels of IL-1β and IL-17A were instead preferentially impacted by the genetic deficiency of dectin-1 (Cunha et al., 2010), highlighting likely different mechanisms through which these variants confer risk to IPA. The production of IL-1β, a critical regulator of early Th17 differentiation (Chung et al., 2009), has been shown to depend on dectin-1 activation (Karki et al., 2015). In turn, dectin-1-mediated signals were reported to affect adaptive immunity to *A. fumigatus* by restraining Th1 responses and enabling Th17 differentiation (Rivera et al., 2011). Taken together, these results confirm the importance of Th17 responses during IPA and highlight the detrimental consequences of genetic deficiency of dectin-1 to their activation.

Despite its exploratory and unbiased nature, our nested case-control study presents however certain limitations. The most relevant regard the inability to conclude about the added benefit of cytokine measurements compared to galactomannan testing or fungal PCR, the definition of cytokine levels as the cause or consequence of the infectious process, and the heterogeneity of the study population regarding the underlying conditions. Finally, the number of patients carrying risk-associated genetic variants do not allow to definitively estimate their effect size on cytokine levels. However, it also raises interesting questions that warrant further investigation. For example, functional studies are required to understand the interrelationships among the identified cytokines and the protective vs. pathophysiological mechanisms underlying IPA, and to identify therapeutic targets amenable to immunomodulation (Carvalho et al., 2012a). In conclusion, our results provide support to additional, well-controlled studies in larger cohorts evaluating whether the diagnostic potential of cytokines, particularly in combination with fungal surrogate markers and integrating the genetic risk profile of the patient, holds clinical value.

## Author contributions

SG, LA-F, CC, and AC designed the study; KL and JM oversaw patient recruitment and collection of clinical specimens and data; SG, CR, CFC, and LB-M performed the laboratory assays; SG, FR, RS, CC, and AC performed the statistical analysis and/or interpreted the data; all authors critically revised and approved the manuscript and are accountable for the accuracy and integrity of the work.

### Conflict of interest statement

The authors declare that the research was conducted in the absence of any commercial or financial relationships that could be construed as a potential conflict of interest.
