# Supplementary material for: Evaluation of Bronchoalveolar Lavage Fluid Cytokines as Biomarkers for Invasive Pulmonary Aspergillosis in At-Risk Patients
Source: Front Microbiol. 2017 Nov 29;8:2362. doi: 10.3389/fmicb.2017.02362 (PMC5712575; doi:10.3389/fmicb.2017.02362)
Supplement: Supplementary file 1 [file Table1.DOCX]

**Table S1. Cytokines in BAL samples from patients with IPA and matched controls.**

| **Cytokine** | **Ctrl** | | | | **IPA** | | | | **P value** |
| --- | --- | --- | --- | --- | --- | --- | --- | --- | --- |
|  | **Median** | **IQR** | **Min** | **Max** | **Median** | **IQR** | **Min** | **Max** |  |
| **EGF** | 64.9 | 25.1 – 164.0 | 6.8 | 2788 | 134.4 | 99.8 – 157.6 | 13.5 | 226.6 | 0.03 |
| **Eotaxin/CCL11** | 14.6 | 7.6 – 22.2 | 5.5 | 35.9 | 18.9 | 9.9 – 29.8 | 3.3 | 34.0 | 0.17 |
| **G-CSF** | 35.0 | 21.8 – 90.6 | 14.0 | 1225 | 57.4 | 23.6 – 128.2 | 5.0 | 214.4 | 0.75 |
| **GM-CSF** | 2.4 | 1.1 – 4.6 | 0.7 | 14.2 | 4.3 | 1.4 – 6.5 | 0.9 | 8.4 | 0.06 |
| **IFNγ** | 2.5 | 1.3 – 5.6 | 1.1 | 21.8 | 3.2 | 2.1 – 5.4 | 1.4 | 10.2 | 0.07 |
| **IL-1α** | 94.0 | 25.7 – 209.6 | 3.1 | 1945 | 98.0 | 18.1 – 1344 | 15.8 | 2504 | 0.29 |
| **IL-1β** | 12.4 | 1.0 – 62.8 | 0.3 | 6748 | 153.7 | 7.8 – 1275 | 0.3 | 11000 | 0.0002 |
| **IL-1Ra** | 183.5 | 49.3 – 372.4 | 29.7 | 2073 | 261.6 | 63.3 – 650.8 | 37.0 | 872.8 | 0.20 |
| **IL-2** | 1.0 | 0.5 – 2.3 | 0.2 | 11.0 | 1.6 | 1.1 – 2.5 | 0.28 | 3.0 | 0.09 |
| **IL-3** | 1.1 | 0.6 – 10.2 | 0.5 | 17.5 | 1.4 | 0.8 – 7.5 | 0.5 | 13.8 | 0.20 |
| **IL-4** | 4.6 | 3.9 – 7.7 | 2.2 | 13.0 | 7.0 | 4.4 – 8.2 | 3.8 | 12.5 | 0.01 |
| **IL-5** | 1.0 | 0.4 – 1.4 | 0.6 | 2.1 | 1.7 | 0.1 – 3.5 | 0.7 | 3.9 | 0.04 |
| **IL-6** | 9.7 | 2.4 – 71.9 | 0.05 | 82.3 | 314.8 | 54.5 – 1058 | 0.8 | 16727 | <0.0001 |
| **IL-7** | 7.1 | 4.2 – 11.3 | 1.7 | 29.8 | 12.0 | 4.1 – 21.4 | 1.6 | 23.8 | 0.40 |
| **IL-8** | 346.7 | 110.7 – 1267 | 39.3 | 11081 | 1952 | 1099 – 4845 | 68.7 | 20500 | <0.0001 |
| **IL-10** | 0.1 | 0.1- 1.4 | 0.1 | 6.4 | 0.4 | 0.1 – 2.9 | 0.1 | 218.2 | 0.13 |
| **IL-12p40** | 10.9 | 3.8 – 30.8 | 2.9 | 45.4 | 17.0 | 9.5 – 23.8 | 3.6 | 33.5 | 0.06 |
| **IL-12p70** | 4.5 | 2.5 – 6.8 | 0.6 | 22.8 | 5.7 | 4.5 – 7.4 | 3.5 | 7.4 | 0.02 |
| **IL-13** | 2.5 | 1.3 – 3.6 | 0.9 | 5.0 | 2.9 | 1.9 – 4.6 | 0.1 | 4.9 | 0.34 |
| **IL-15** | 3.1 | 1.2 – 5.4 | 0.6 | 19.7 | 4.0 | 3.5 – 5.8 | 2.6 | 9.8 | 0.01 |
| **IL-17A** | 0.01 | 0.01 – 0.4 | 0.01 | 7.0 | 1.9 | 0.4 – 6.2 | 0.01 | 34.7 | <0.0001 |
| **IL-18** | 10.2 | 2.5 – 33.0 | 0.1 | 119.7 | 13.9 | 3.0 – 76.5 | 0.1 | 1386 | 0.35 |
| **IL-21** | 1.0 | 0.7 – 2.0 | 0.2 | 9.4 | 1.4 | 0.9 – 2.0 | 0.2 | 2.1 | 0.24 |
| **IL-22** | 19.9 | 10.7 – 32.7 | 3.0 | 53.9 | 31.7 | 16.6 – 44.8 | 5.0 | 61.0 | 0.02 |
| **IL-23** | 56.6 | 25.2 – 99.4 | 4.0 | 444.9 | 156.4 | 102.1 – 305.9 | 23.6 | 2181 | <0.0001 |
| **IP-10/CXCL10** | 27.4 | 12.0 – 332.0 | 5.5 | 3867 | 79.9 | 12.4 – 123.6 | 8.7 | 5707 | 0.67 |
| **MCP-1/CCL2** | 223.3 | 107.9 – 535.8 | 2.7 | 7445 | 740.6 | 25.0 - 1419 | 2.6 | 5208 | 0.15 |
| **MIP-1α/CCL3** | 30.0 | 21.1 – 56.2 | 15.8 | 518.6 | 53.0 | 19.1 – 75.9 | 17.5 | 126.0 | 0.09 |
| **MIP-1β/CCL4** | 29.2 | 12.4 – 324.4 | 5.8 | 324.4 | 61.4 | 7.2 – 124.9 | 5.5 | 221.9 | 0.07 |
| **TNFα** | 0.1 | 0.02 – 1.98 | 0.02 | 320.5 | 3.8 | 1.1 – 14.9 | 0.02 | 1925 | <0.0001 |
| **TNFβ** | 1.4 | 0.9 – 3.0 | 0.5 | 4.8 | 2.1 | 1.2 – 3.5 | 0.3 | 4.1 | 0.17 |
| **VEGF** | 1892 | 1101 – 4256 | 350.0 | 11250 | 2500 | 1182 – 6250 | 671.5 | 27500 | 0.41 |

Results are expressed as pg/mL. Ctrl, control; IPA, invasive pulmonary aspergillosis; IQR, interquartile range; Min, minimum; Max, maximum; EGF, epidermal growth factor; CCL, C-C motif chemokine; G-CSF, granulocyte colony-stimulating factor; GM-CSF, granulocyte macrophage colony-stimulating factor; IFN, interferon; IL, interleukin; IL-1Ra, IL-1 receptor antagonist; IP-10, IFNγ-induced protein 10; CXCL, C-X-C motif chemokine; MCP-1, monocyte chemoattractant protein-1; MIP, macrophage inflammatory protein; TNF, tumor necrosis factor; VEGF, vascular endothelial growth factor. P values are for the Student’s t-test or the non-parametric Mann-Whitney *U* test, following the Shapiro-Wilk normality test. After adjustment for multiple testing with the use of the Bonferroni’s method, the level for significance was defined as P=0.0016 (based on 32 independent tests).
