# Supplementary material for: Evaluation of Bronchoalveolar Lavage Fluid Cytokines as Biomarkers for Invasive Pulmonary Aspergillosis in At-Risk Patients
Source: Front Microbiol. 2017 Nov 29;8:2362. doi: 10.3389/fmicb.2017.02362 (PMC5712575; doi:10.3389/fmicb.2017.02362)
Supplement: Supplementary file 2 [file Table2.DOCX]

**Table S2. Cytokines in serum samples from patients with IPA and matched controls.**

| **Cytokine** | **Ctrl** | | | | **IPA** | | | | **P value** |
| --- | --- | --- | --- | --- | --- | --- | --- | --- | --- |
|  | **Median** | **IQR** | **Min** | **Max** | **Median** | **IQR** | **Min** | **Max** |  |
| **IL-1β** | n.d. | n.d. | n.d. | n.d. | n.d. | n.d. | n.d. | n.d. | n.d. |
| **IL-6** | 5.1 | 1.1 – 20.6 | 0.2 | 320.1 | 44.9 | 10.5 – 100.7 | 0.3 | 2908 | 0.01 |
| **IL-8** | 11.22 | 4.5 – 35.1 | 0.24 | 171.8 | 81.7 | 16.5 – 331.7 | 8.1 | 3734 | 0.003 |
| **IL-17A** | 0.02 | 0.02 – 002 | 0.02 | 0.02 | 0.02 | 0.03 – 0.34 | 0.02 | 1.6 | 0.005 |
| **IL-23** | 3 .0 | 3.0 – 3.8 | 3.0 | 24.5 | 4.1 | 3.0 – 24.5 | 3.0 | 214.4 | 0.04 |
| **TNFα** | 0.1 | 0.02 – 2.9 | 0.02 | 13.1 | 0.02 | 0.02 – 1.2 | 0.02 | 8.1 | 0.5 |

Results are expressed as pg/mL. Ctrl, control; IPA, invasive pulmonary aspergillosis; IQR, interquartile range; Min, minimum; Max, maximum; n.d., not detectable; IL, interleukin; TNF, tumor necrosis factor.
