# Supplementary material for: Evaluation of Bronchoalveolar Lavage Fluid Cytokines as Biomarkers for Invasive Pulmonary Aspergillosis in At-Risk Patients
Source: Front Microbiol. 2017 Nov 29;8:2362. doi: 10.3389/fmicb.2017.02362 (PMC5712575; doi:10.3389/fmicb.2017.02362)
Supplement: Supplementary file 3 [file Table3.DOCX]

**Table S3. Comparison of net reclassification index values for alveolar cytokines according to risk genotypes.**

| **Genetic variant** | **Genotype** | **Cytokine cut-off†** | **ΔSe** | **Δ(1-SP)** | **NRI** |
| --- | --- | --- | --- | --- | --- |
| ***PTX3* rs2305619** | **A+** | **IL-6 ≥88.9** | -0.02 | 0.06 | 0.62 |
|  | **GG** |  | -0.09 | -0.29 | 0.17 |
|  | **A+** | **IL-8 ≥904** | -0.02 | -0.06 | 0.58 |
|  | **GG** |  | 0.23 | 0.10 | 0.50 |
| ***CLEC7A* rs16910526** | **TT** | **IL-1β ≥27.1** | 0.02 | -0.01 | 0.31 |
|  | **TG** |  | -0.13 | -0.12 | 0.20 |
|  | **TT** | **IL-17A ≥0.66** | 0.00 | -0.02 | 0.51 |
|  | **TG** |  | 0.05 | -0.14 | 0.47 |

†Cut-off values of cytokines are expressed as pg/mL. The A+ category indicates combined AA and AG genotypes. ΔSe, change in sensitivity; Δ(1-SP), change in 1-specificity; NRI, net reclassification index.
