# Supplementary figures and images for: Evaluation of Bronchoalveolar Lavage Fluid Cytokines as Biomarkers for Invasive Pulmonary Aspergillosis in At-Risk Patients
Source: Front Microbiol. 2017 Nov 29;8:2362. doi: 10.3389/fmicb.2017.02362 (PMC5712575; doi:10.3389/fmicb.2017.02362)

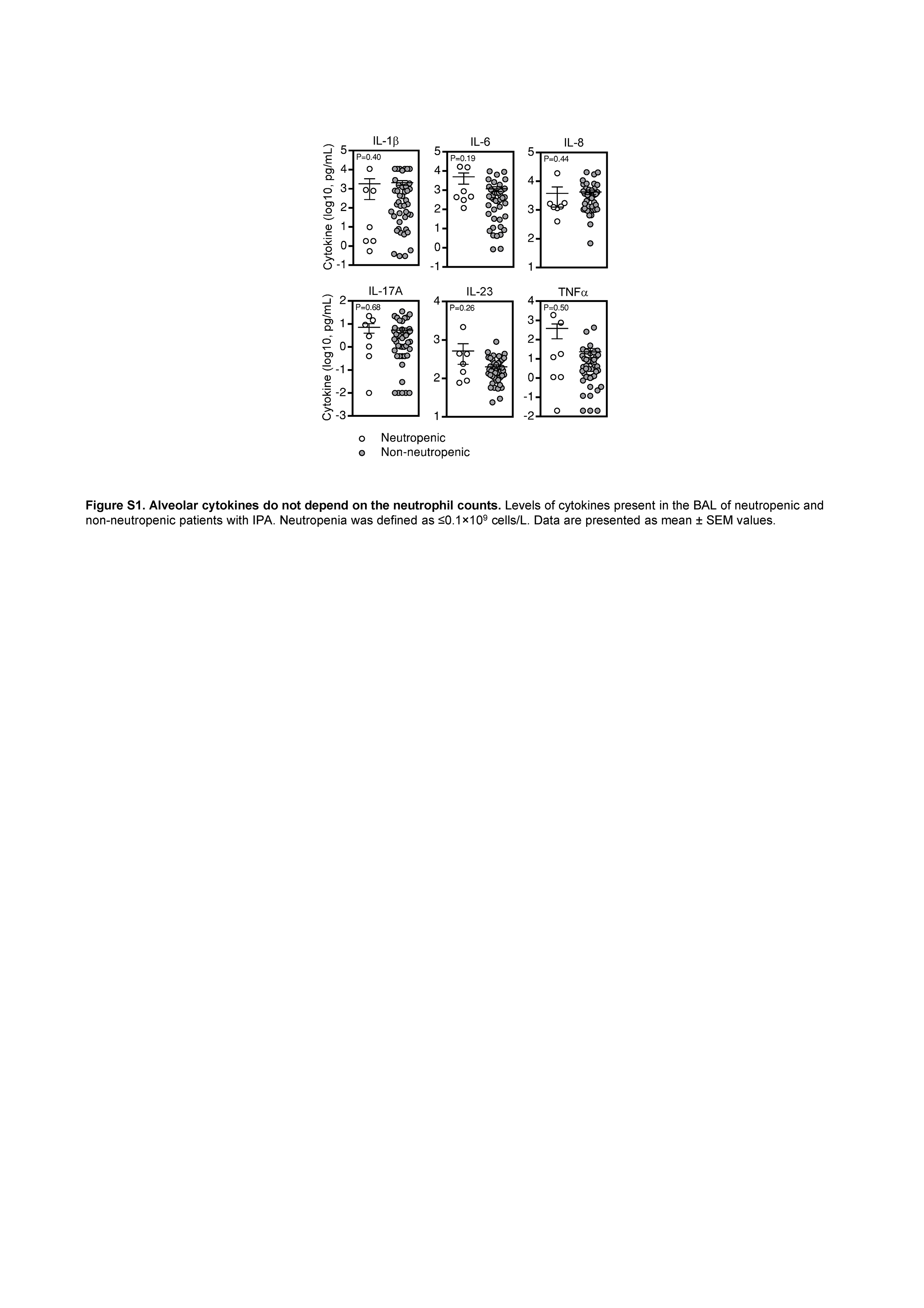

Supplement: Supplementary file 4 [file Image1.TIFF]

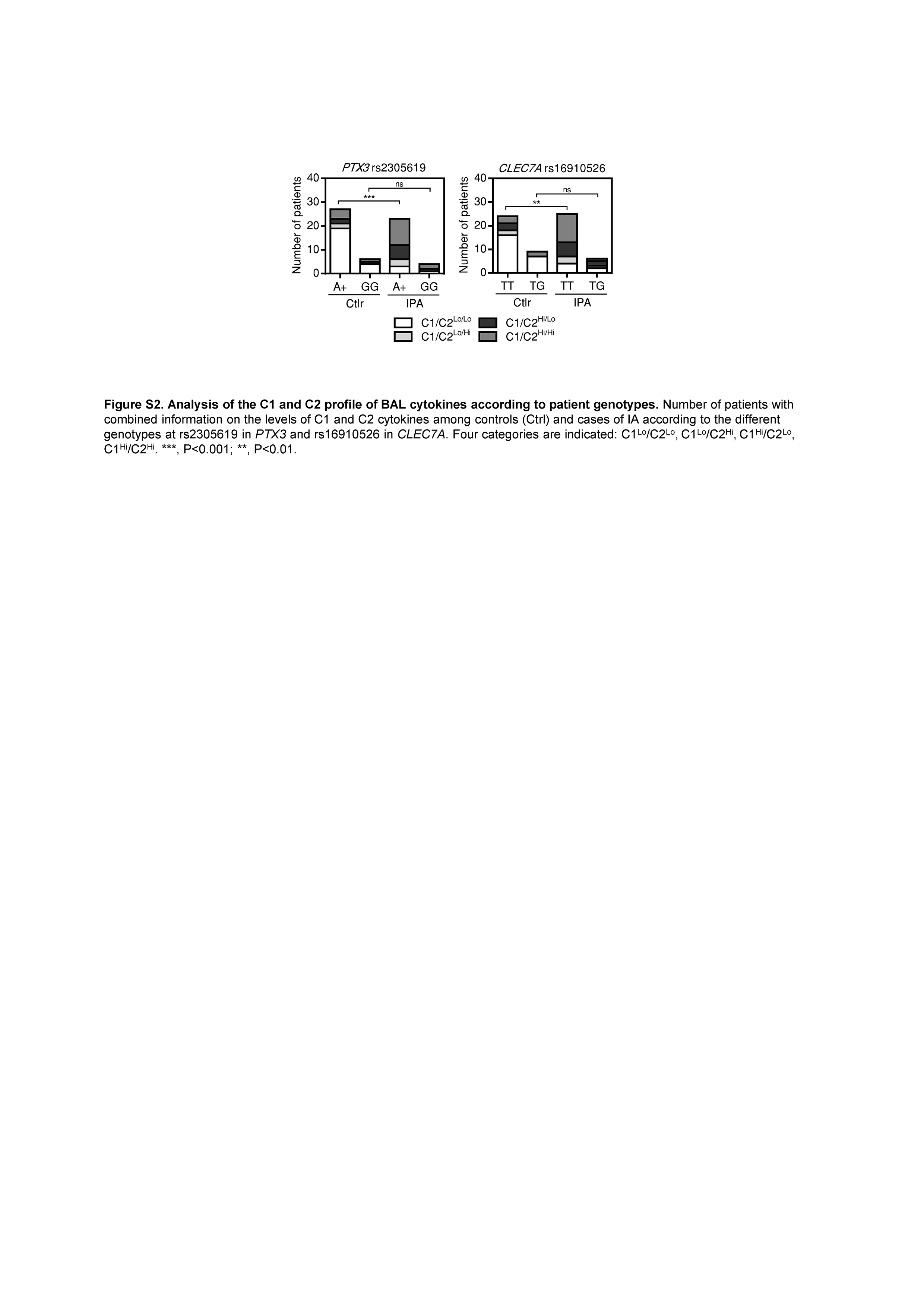

Supplement: Supplementary file 5 [file Image2.TIFF]
